# Physical and Psychoacoustic Characteristics of Typical Noise on Construction Site: “How Does Noise Impact Construction Workers’ Experience?”

**DOI:** 10.3389/fpsyg.2021.707868

**Published:** 2021-07-28

**Authors:** Xinhao Yang, Yitong Wang, Ruining Zhang, Yuan Zhang

**Affiliations:** ^1^Eco-Building Physics Technology and Evaluation Provincial Key Lab, School of Architecture and Urban Planning, Shenyang Jianzhu University, Shenyang, China; ^2^Railway No.9 Bureau Group 4th Engineering Co., Ltd., Shenyang, China

**Keywords:** construction workers, construction noise, noise level, physical and psychoacoustic characteristics, sound annoyance, impact on hearing, impact on on-site communication

## Abstract

Construction noise is an integral part of urban social noise. Construction workers are more directly and significantly affected by construction noise. Therefore, the construction noise situation within construction sites, the acoustic environment experience of construction workers, and the impact of noise on them are highly worthy of attention. This research conducted a 7-month noise level (L_Aeq_) measurement on a construction site of a reinforced concrete structure high-rise residential building in northern China. The noise conditions within the site in different spatial areas and temporal stages was analyzed. Binaural recording of 10 typical construction noises, including earthwork machinery, concrete machinery, and hand-held machinery, were performed. The physical acoustics and psychoacoustic characteristics were analyzed with the aid of a sound quality analysis software. A total of 133 construction workers performing 12 types of tasks were asked about their subjective evaluation of the typical noises and given a survey on their noise experience on the construction site. This was done to explore the acoustic environment on the construction site, the environmental experience of construction workers, the impact of noise on hearing and on-site communications, and the corresponding influencing factors. This research showed that the noise situation on construction sites is not optimistic, and the construction workers have been affected to varying degrees in terms of psychological experience, hearing ability, and on-site communications. Partial correlation analysis showed that the construction workers’ perception of noise, their hearing, and their on-site communications were affected by the noise environment, which were correlated to varying degrees with the individual’s post-specific noise, demand for on-site communications, and age, respectively. Correlation analysis and cluster analysis both showed that the annoyance caused by typical construction noise was correlated to its physical and psychoacoustic characteristics. To maintain the physical and mental health of construction workers, there is a need to improve on the fronts of site management, noise reduction, equipment and facility optimization, and occupational protection.

## Introduction

In the process of urbanization, cities are often occupied by a large number of construction sites, which has caused the problem of construction noise. Although most construction noise is not continuous, its high sound pressure level and overall long exposure time have caused some problems to surrounding residents. In China, the number of complaints about construction noise ranked first among all types of noise in 2019, accounting for 45.4% of all complaints (China Ministry and Environment, 2020). The harm of noise to the human body cannot be ignored. As the most persistent physical contaminant in the human environment, a large number of empirical studies have focused on the adverse effects of noise on individual health. Such effects included hearing impairment (Clark and Paunovic, 2018), cardiovascular disease (Van Kempen et al., 2018), sleep disorders, etc. (Jung et al., 2020). In contrast, construction workers within construction sites are more directly and more significantly affected by construction noise, whose occupational noise exposure also deserves attention. As of 2019, there were 54.27 million workers in the construction industry in China (China National Bureau of Statistics, 2020). Most of the workers were disturbed by noise, which was caused by both the construction actions and the machinery. In the field of occupational noise, noise-induced hearing loss (NIHL) is the most direct consequence to workers, and has become the focus of extensive research (Basner et al., 2014). It was estimated that 16–24% of hearing impairment was related to works in the world (Nelson et al., 2005). Long term high-exposure noise could cause NIHL and even cause permanent hearing losses (ISO, 2013). Statistics had shown that more than 20 million Americans worked with high-exposure noise (Tak et al., 2010).

The academic community generally used equivalent continuous A-weighted sound pressure level (L_Aeq_) as an evaluation index of noise exposure (Neitzel and Fligor, 2017). In the temporal dimension, 24 h has usually been used for environmental noise exposure and 8 h has generally been used for occupational noise exposure. At the same time, occupational noise exposure has been quantified by the exchange rate (ER), which was the change in average noise level (in dB). This corresponded to doubling or halving the allowed exposure time. Most countries and organizations used 3 dB as the basis for noise level changes, namely L_EX_, also known as L_*A*8hn_ or L_EX8h_ (Neitzel and Fligor, 2017). Based on this, The National Institute for Occupational Safety and Health (NIOSH) proposed the Recommended Exposure Limit (REL). Among these parameters, L_EX_ is 85 dB(A) and ER is set to 3 dB (NIOSH, 1998). The American Conference of Governmental Industrial Hygienists (ACGIH) proposed the Threshold Limit Value (TLV) (ACGIH, 1999), which was essentially the same as NIOSH. Its purpose was to protect the relevant workers after 40 years of Occupational noise exposure. The median of their hearing loss was less than 2 dB. In addition, the European Union (European Union, 2003), Japan (Shaikh, 1999), and China (China National Standard, 2008) have also established occupational exposure restriction mechanisms. Its content was similar to the relevant regulations by the NIOSH and the ACGIH. However, the reality was not optimistic. According to the construction noise monitoring results in different countries and regions, the construction noise all exceeded the specification limit to varying degrees (Neitzel et al., 1999, 2011; Leensen et al., 2011; Haron et al., 2014). This poses potential threats to surrounding people and construction workers.

High-exposure noise will bring negative auditory feelings to construction workers. Related researches were devoted to exploring the relationship between construction noise and noise-induced annoyance. Noise-induced annoyance is defined as an individual’s adverse reaction to noise (ISO, 2003), including dissatisfaction, bother, annoyance, and disturbance due to noise (Guski et al., 1999). Most of these studies conducted researches on urban residents through questionnaires (Chunk, 2000; Darus et al., 2015; Liu et al., 2017), and the results focused on the relationship between types of noise or noise level and noise-induced annoyance. However, not only the SPL, but also different construction noise types had different acoustic characteristics, and their subjective perceptions were also different (Lee et al., 2015). In addition to physical acoustics, psychoacoustics is equally important. Psychoacoustic indicators are mainly used in acoustic measurements, sound quality exploration, subjective prediction, etc. The areas involved include car sound quality (Lee, 2008; Volandri et al., 2018), traffic noise (Raggam et al., 2007), mechanical noise (Dragonetti et al., 2017), household appliance noise (Jin et al., 2007), soundscape (Aletta et al., 2020), etc. They were aimed at people’s subjective feelings about sound. Zwicker proposed the psychoacoustic annoyance model, whose indicators included the percentile Loudness (N_5_), Sharpness (S), Fluctuation strength (F), and Roughness (R). This model was proved to be suitable for different types of noise-induced annoyance estimation (Zwicker and Fastl, 1999). In the field of construction noise, the exploration of psychoacoustic indicators was gradually carried out (Carletti, 2013; Lee et al., 2015), which was also mainly used to explore the relationship between construction noise and noise-induced annoyance.

In order to reduce the risk of workers being exposed to harmful health factors at work, it is the goal of occupational health to put and maintain workers in an occupational environment that adapts to their physical and psychological abilities (Alli, 2008). As the direct contact of construction noise, the physical and psychological changes of construction workers affected by this are worthy of attention.

Based on this, this research carried out empirical studies through noise level measurement of the construction site, questionnaire of construction workers, collection of typical construction noise and acoustic analysis. This research aimed to explore the experience and impact of construction workers on the acoustic environment of the construction site and typical construction noise. Furthermore, this research also explored the factors governing the relevance between the experience of construction noise and the hearing ability and communication between construction workers. This was conducted using statistical methods such as correlation analysis and principal components analysis (PCA).

## Materials and Methods

### Noise Level Measurement of the Construction Site

**FIGURE 1:**
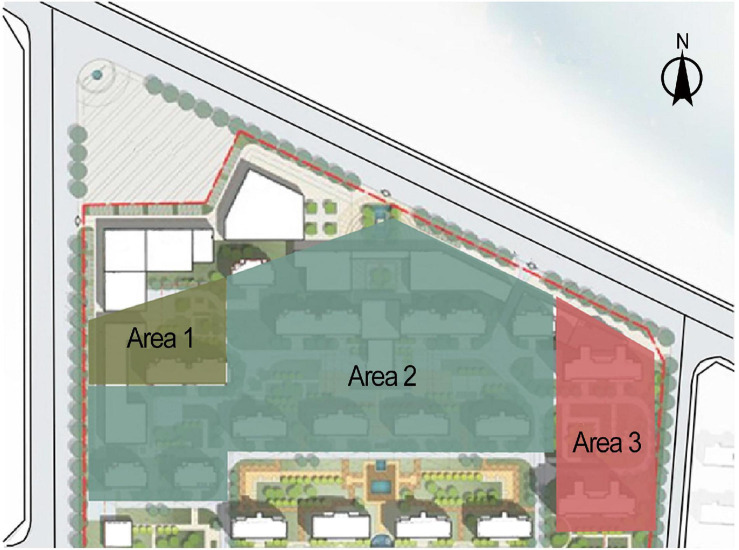
Distribution of the three areas in the site.

This study was based on a construction site of a residential complex in Shenyang, China. The site covers a total area of about 25,500 m^2^ and includes two construction areas (area 1, area 2) and a living area for workers (area 3) (Figure 1). There are no facilities separating the three areas. The overall construction period for the 12 buildings with reinforced concrete shear wall structures on the site was 24 months. A 7-month delay was present between the start of the two construction areas. There were three construction stages measurement for the purpose of noise level monitoring: earthworks, concrete framing and block masonry, and indoor structuring.

**FIGURE 2:**
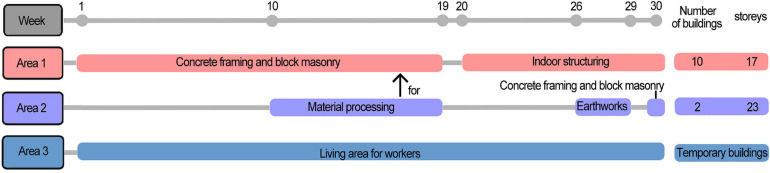
During the 30-week measurement, the different construction stages in the three areas.

The site was divided into 270 square grids of side length 10 m. The position occupied by the main building was removed, and so 184 grids were actually measured. Two surveyors performed L_Aeq_ measurement for 30 s at the center point of each grid from 10:00 to 11:00 for one working day per week. The measuring tool was a B&K 2250 sound level meter with a measuring height of 1.5 m. The overall measurement lasted for 30 weeks (2019.04.05–2019.10.17). There were no measurements in the week of 6, 22, 24, and 28, due to site and weather conditions, and thus a total of 26 measurements were taken. Except for the grid points that were restricted by on-site operating conditions, a total of 4539 valid data were obtained for the 26 weeks. Figure 2 shows the construction information of each area in the measurement stages.

### Typical Noise Collection and Analysis

As the main source of construction noise, the noise created by construction machineries in large dictate the overall noise level within the construction site. In order to explore the acoustic characteristics of mechanical noise, 10 typical noise sources (Table 1) including earthwork machinery, concrete machinery, and small hand-held machinery were selected for binaural acoustic measurement at the construction site (Brüel & Kjaer 4101A). The measurement height was 1.5 m and external noises were kept minimum during the measurements. The recording time of each type of noise was 2–3 min with normal operation of each machine, and the background noise during recording was also measured. Audio editing was carried out through B&K Connect sound quality analysis software. Based on the principle of intercepting the complete characteristic period, 10 s samples were intercepted from the material for physical and psychoacoustic analysis. Physical acoustic indicators include L_Aeq_, L_5_, L_95_, and psychoacoustic indicators include Loudness (N) (ISO, 2017), Fluctuation strength (F), Roughness (R), Sharpness (S) (Zwicker and Fastl, 1999). The perceived overall Loudness of a time-variant sound is well represented by the percentile Loudness N_5_ (ISO, 2018), which is the peak Loudness within a certain timeframe. In addition, other psychoacoustic percentile parameters should also be considered to characterize statistical eigenvalues of different psychoacoustic indicators in the temporal domain (ISO, 2019). In addition, the relevant indicators have been applied to the optimization of mechanical noise (Carletti and Pedrielli, 2011) and residents’ perception of soundscape in urban public space (Rychtarikova et al., 2008; Rychtarikova and Vermeir, 2013). Thus, N_5_, F_10_, R_10_, and S_5_ should also be calculated. The arithmetic average of the data from both channels in the binaural acoustic measurement system was taken for all of the above parameters.

**TABLE 1:** Ten types of typical noise sources, sound-producing machinery, annual presence time, operator types, and on-site measurement distance.

**Machinery type**	**Construction noise type**	**Machinery sound**	**Duration on site (months/year)**	**Operating worker type**	**Measurement distance**
Earthwork machinery	Earthwork transportation	Dump truck	6	Machinery operator	1–3 m
		Loader	3	Machinery operator	
	Earthwork crushing	Breaker	1	Machinery operator	
	Earthwork excavation	Excavator	3	Machinery operator	
Concrete machinery	Concrete pumping	Concrete pump	8	Machinery operator	1.5–2 m
	Concrete vibration	Concrete vibrator	8	Laborer	
Hand machinery	Material sanding	Angle grinder	12	Laborer/Plumber/Bricklayer	1.5–2 m
	Surface crushing	Jackhammer	12	Carpenter/Laborer	
	Material drilling	Electric drill	12	Laborer/Electric engineer/Plumber	
	Screw installation	Electric screwdriver	12	Electric engineer/Plumber	

### Acoustic Environment Experience and Impact Survey of Construction Workers

#### Questionnaire Design and Data Collection

In order to understand the acoustic environment experience of the workers during the construction period and explore its influencing factors, a questionnaire was conducted for the workers. The questionnaire was divided into four parts: noisiness level of construction, self-evaluation on hearing ability (SEHA) and evaluation of the interference on on-site communications (EIOSC), evaluation of the annoyance by typical noises, and individual characteristics (Table 2). Among them, a five-level descriptive rating scale was employed for the noisiness level of construction and the evaluation of typical sound source annoyance, and the SEHA and EIOSC used a seven-level descriptive rating scale. All respondents signed an informed consent form before completing the questionnaire. They were informed of the purpose of the study and the use of the data. Ethical review and approval was not required for this study with the local legislation and institutional requirements. A total of 143 questionnaires including 12 types of tasks were distributed, and 133 valid questionnaires were returned, with a recovery rate of 93.0%.

**TABLE 2:** Questionnaire composition.

**Variable**	**Question description**	**Type of response**
Self-evaluation on hearing ability (SEHA)	What do you think of the impact of construction noise on your own hearing since you participated in site work?	Descriptive rating scale: 1–7 (1-No impact at all to 7-Extremely high impact)
Evaluation of the Interference on On-Site Communications (EIOSC)	What do you think of the impact of construction noise on your communication with others?	
Noisiness level	How noisy do you think the overall acoustic environment of the construction site is?	Descriptive rating scale: 1–5 (1- NOT noisy at all to 5 Extremely noisy)
Typical construction noise annoyance	Please evaluate the annoyance level of the said machinery (The 10 machineries listed in table 1)	Descriptive rating scale: 1–5 (1- Not annoying at all to 5- Extremely annoying)
Individual characteristics	Age, Gender, Type of tasks, Working years	

#### Evaluation on the Post-specific Noise Level and Demand for Communication

In the form of a focus-group, four project managers who were familiar with the conditions of the construction site were invited to conduct a professional evaluation of the post-specific noise level and the demand for on-site communications level of the construction site for each type of workers. The evaluation index was a nine-level numerical level.

### Statistical Analysis

To eliminate the effect of other variables, partial correlation analysis was employed to investigate the relationship between the results of the self-evaluation of the workers and the post-specific noise, the demand for on-site communications, age and working years. In addition, principal components analysis was used to cluster the ten typical noises according to the 133 respondents’ evaluation of the annoyance by typical noises. The Jonckheere–Terpstra test was subsequently used to investigate common features within each cluster of noises.

## Results

### The Noise Level in the Construction Site

The 4539 measured SPL data (L_Aeq_) were in the range of 51.0–112.0 dB(A). These data were compared with the occupational noise exposure standards. The results showed that among the 4539 data points, the limit values set by various countries and institutions were exceeded to varying degrees (Table 3). 18.6% of the data points exceeded the limit of L_EX_ = 80 dB(A), and 4.3% exceeded the limit of L_ex_ = 85 dB(A).

**TABLE 3:** Occupational noise exposure limit and the proportion of over-limit samples (*N* = 4539).

**Exposure duration (h)**	**China**		**American**						**European Union Directive 2003/10/EC**					
	**GB/T 12801-2008**		**ACGIH**		**NIOSH**		**OSHA**		**Lower exposure action value**		**Upper exposure action value**		**Exposure limit**	
	**L_EX_ dB(A)**	**Out of limit (%)**	**L_EX_ dB(A)**	**Out of limit (%)**	**L_EX_ dB(A)**	**Out of limit (%)**	**L_EX_ dB(A)**	**Out of limit (%)**	**L_EX_ dB(A)**	**Out of limit (%)**	**L_EX_ dB(A)**	**Out of limit (%)**	**L_EX_ dB(A)**	**Out of limit (%)**
8	85	4.3	85	4.3	85	4.3	90	1.5	80	18.6	85	4.3	87	2.6
4	88	2.0												
2	91	1.3	88	2.0	88	2.0	95	0.4	83	10.2	88	2.0	88	2.0
1	94	0.4	91	1.3	91	1.3	100	0.2	86	3.5	91	1.3	91	1.3
1/2	97	0.2	94	0.4	94	0.4	105	0.1	89	1.6	94	0.4	94	0.4
1/4	100	0.2												
1/8	103	0.1												
0	115	0.1					115	0.1						

To compare the noise levels of different construction stages in the construction site, five measurements from different construction stages were selected for comparison among 26 measurements. Through the Arc GIS platform, corresponding attributes were given to the spatial coordinate elements of the construction site, and the Kriging interpolation method was used to produce a noise map of the site.

**FIGURE 3:**
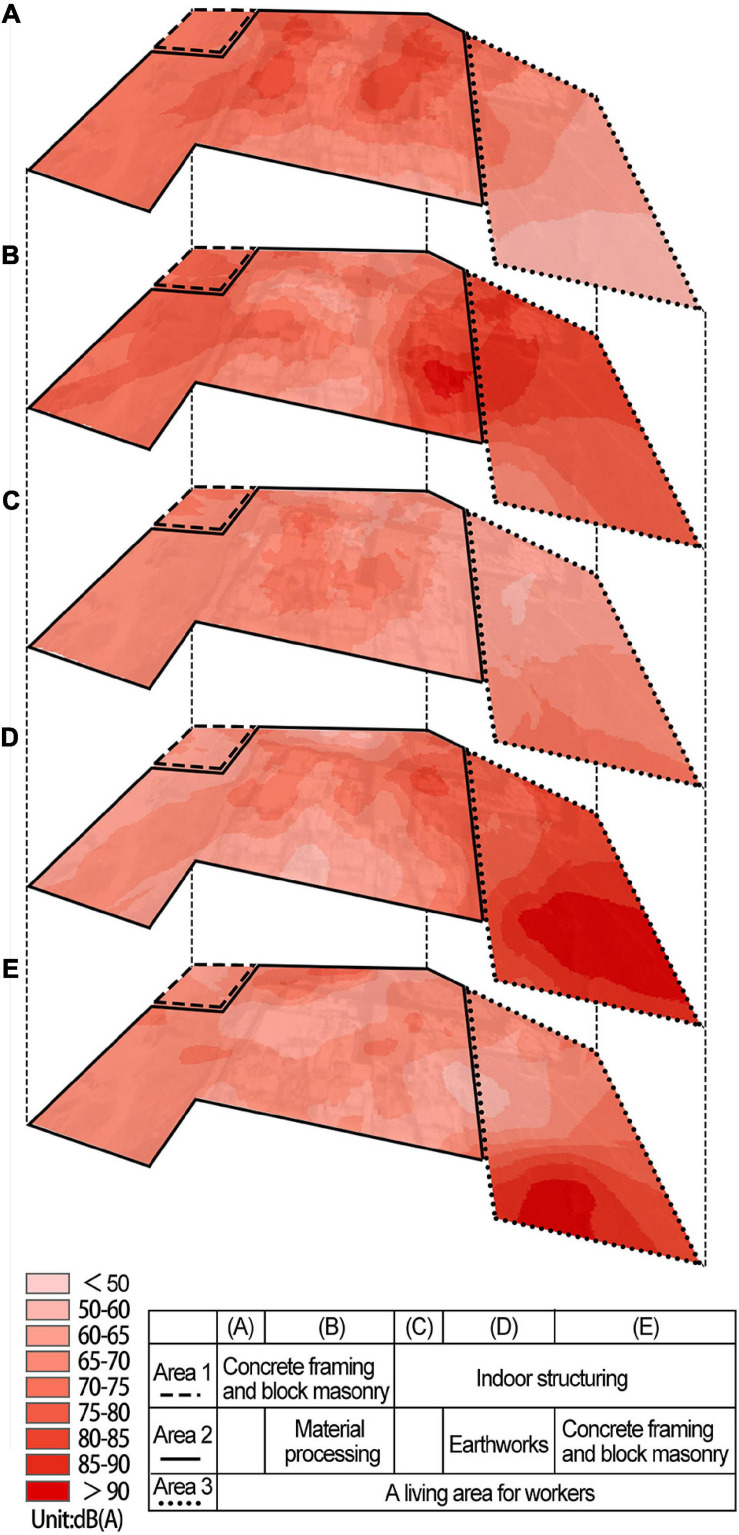
Noise levels of different construction stages: **(A)** 26th Apr, 2019; **(B)** 19th Jun, 2019; **(C)** 22th Aug, 2019; **(D)** 20th Sep, 2019; **(E)** 19th Oct, 2019.

The noise levels of different construction stages were not consistent (Figure 3). In terms of SPL: earthworks > concrete framing and block masonry > indoor structuring. Earthworks included many large-scale mechanical types of equipment, such as excavators, loaders, dump trucks, etc., which would produce a higher SPL during operation and the duration of the noise would be longer. The concrete framing and block masonry stage used more concrete machinery and hand-held machinery, and the SPL produced was smaller than that of the earthwork machinery. In the indoor structuring stage, most of the construction work was transferred to the building interior, which had a barrier effect on the sound transmission, resulting in the minimum contribution to the noise within the site. It is worth noting that area 2 (See Figure 3B) was the material processing area, and the rebar cutter and steel bar bender used would produce a relatively high SPL. In general, the distribution of construction noise within the site was not uniform. In addition, the sound pressure levels produced by different construction stages were different. Construction noise was mainly concentrated in the area where the construction work was carried out. Therefore, the occupational noise protection of related work types is worthy of attention.

### Acoustic Environment Experience and Impact

#### Perceived Noisiness Level of the Environment and Self-Evaluated Impact on Workers

The basic characteristics for the composition of respondents included gender, age, working years, and types of tasks (Table 4). The reliability of the variables in the questionnaire was tested, and the results showed that Cronbach α was 0.716. This was good reliability that allowed the next step of data analysis to be carried out.

**TABLE 4:** The basic characteristics for the composition of respondents.

**Gender (N%)**		**Age (N%)**					**Working years (N%)**				
**Male**	**Female**	**≤30**	**31–35**	**36–40**	**41–45**	**≥46**	**≤5**	**6–10**	**11–15**	**16–20**	**≥21**
124 (93.2)	9 (6.8)	27 (20.3)	38 (28.6)	24 (18.0)	27 (20.3)	17 (12.8)	18 (13.5)	28 (21.1)	35 (26.3)	26 (19.5)	26 (19.5)
**Types of tasks (N%)**											
**Project manager**	**Logistics and service staff**	**Rebar worker**	** Carpenter**	**Bricklayer**	**Machinery operator**	**Laborer**	**Scaffolder**	**Plumber**	**Electric engineer**	**Door and window installer**	**Welder**
10 (7.5)	6 (4.5)	14 (10.5)	15 (11.3)	19 (14.3)	8 (6.0)	10 (7.5)	10 (7.5)	12 (9.0)	13 (9.8)	7 (5.3)	9 (6.8)

*N = 133.*

**FIGURE 4:**
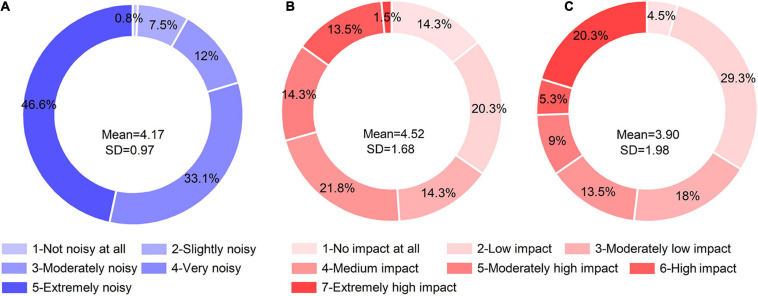
Results of noisiness level and self-evaluation: **(A)** Noisiness; **(B)** SEHA; **(C)** EIOSC.

The result showed that only one respondent thought that the construction site was not noisy (Figure 4). The respondents who thought the site was “Very noisy” and “Extremely noisy” accounted for 33.1 and 44.6%, respectively. This meant that the respondents generally thought that the construction site was noisy (mean = 4.17), and the evaluation was consistent (SD = 0.97).

In the results of the SEHA and EIOSC, 29.3% (Mean = 4.52, SD = 1.68) and 34.6% (Mean = 3.90, SD = 1.98) of the respondents believed that the impact of noise on their hearing ability and on-site communications has been “Moderately high impact” or higher, respectively. The two self-evaluation results were highly discrete, which indicated that the subjective evaluation was also affected by other factors.

In order to explore the factors for noisiness level and self-evaluation results, the three variables were put to perform partial correlation analysis with post-specific noise, demand for on-site communications, age, and working years.

There was a significant positive correlation between the noisiness level of the construction and the post-specific noise [*r*_(133)_ = 0.497, *p* = 0.000] (Table 5). It can be seen that the respondents’ results of noisiness level were affected by the post-specific noise.

**TABLE 5:** Results of partial correlation in evaluation results and related variables.

**Variable**		**Post-specific noise**	**Demand for on-site communications**	**Age**	**Working years**
Noisiness level	Partial correlation	0.497**	–0.013	0.062	–0.086
	Sig. (2-tailed)	0.000	0.879	0.480	0.332
SEHA	Partial correlation	0.538**	−0.357**	0.028	0.126
	Sig. (2-tailed)	0.000	0.000	0.752	0.154
EIOSC	Partial correlation	0.604**	−0.312**	0.192*	–0.116
	Sig. (2-tailed)	0.000	0.000	0.028	0.188

*Correlation is significant at the 0.05 level (2-tailed).

***Correlation is significant at the 0.01 level (2-tailed).

The post-specific noise level [*r*_(133)_ = 0.538 *p* = 0.000] had different effects on the SEHA. In other words, workers believed that post-specific noise affected their hearing ability. It is worth mentioning that demand for on-site communications [*r*_(133)_ = −0.357 *p* = 0.000] was negatively correlated with impact on the hearing ability, and could also be related to the adaptability of workers on the construction site.

The EIOSC was significantly negatively correlated with the post-specific noise [*r*_(133)_ = 0.604, *p* = 0.000] and age [*r*_(133)_ = 0.192 *p* = 0.028]. It meant the post-specific noise and the hearing loss due to age had a significant impact on on-site communications. At the same time, it was found that EIOSC was negatively correlated with the demand for on-site communication [*r*_(133)_ = −0.312 *p* = 0.000], and could also be related to the adaptability of workers on the construction site.

### Acoustic Characteristics and Annoyance Analysis of Typical Construction Noise

#### Analysis of the Acoustic Characteristics

**FIGURE 5:**
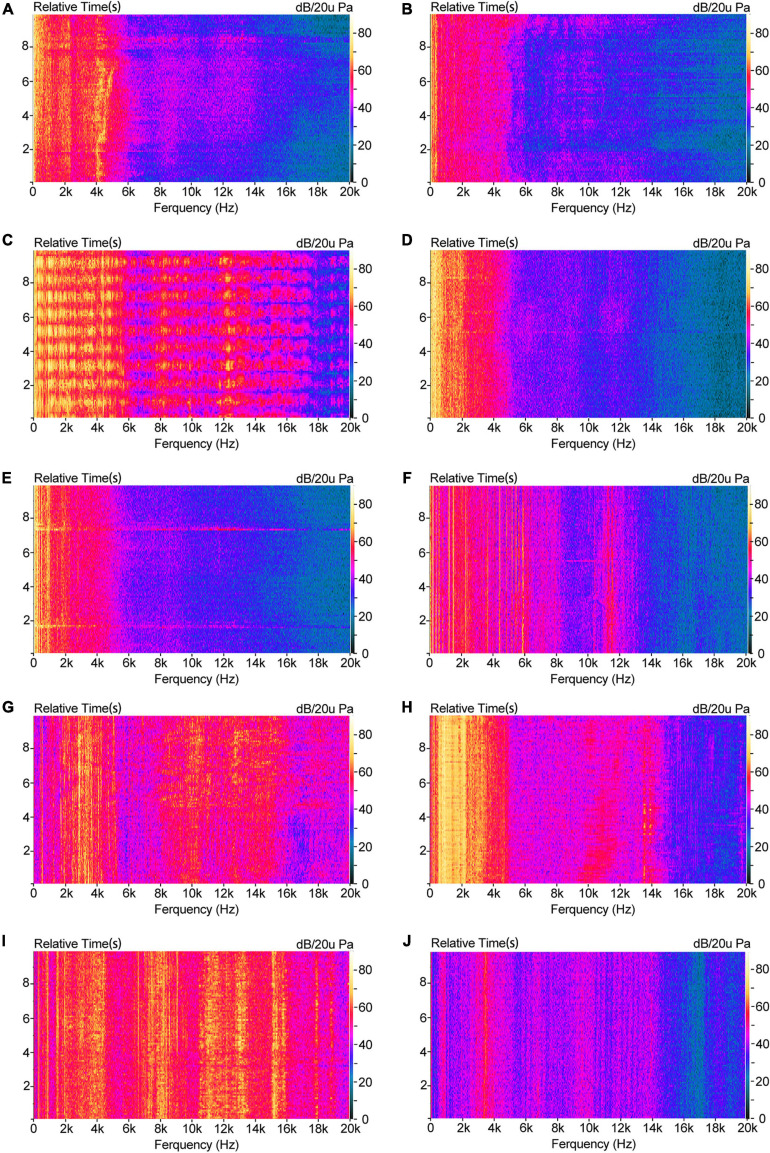
FFT vs. Time: **(A)** Dump truck; **(B)** Excavator; **(C)** Breaker; **(D)** Loader; **(E)** Concrete pump; **(F)** Concrete vibrator; **(G)** Angle grinder; **(H)** Jackhammer; **(I)** Electric drill; **(J)** Electric screwdriver.

Figure 5 shows the frequency and energy distribution of the 10 typical construction noise over a 10 s period. As the interaural level difference was typically very small (Kang et al., 2018), data from the left channel was arbitrarily chosen to conduct the analysis. It can be seen from the frequency domain distribution characteristics of energy that the loader, the dump truck, and the excavator had higher energy in the low, medium, and high frequency (within 6 kHz) regions. The angle grinder, the jackhammer and the electric drill were distributed at even higher frequency region. The electric screwdriver had a more obvious energy concentration around 4 kHz. From the perspective of temporal-domain characteristics, the impact sound generated by the concrete pump and the breaker had strong periodic characteristics due to their operation mode. The time period of the former was about 1 s, and the latter was about 5 s. The remaining sounds showed a certain steady-state in 10 s.

The physical and psychoacoustic indices of the ten typical construction noises are shown in Table 6. The table also contains the maximum, minimum, mean, and standard deviation values. The L_Aeq_ of the ten kinds of construction noise was between 75.82 and 93.87 dB(A). Among them, that generated by the jackhammer and the breaker were above 90 dB(A), that generated by the electric screwdriver and the excavator were below 80 dB(A), and the generated by the other machinery was between 80 and 90 dB(A). Except for the dump truck, which was recorded on the move, the SPL fluctuations (L_5_–L_95_) were relatively small due to the stable sound of the intercepted audio samples. In terms of the psychoacoustic index, the breaker had the highest Loudness, reaching 98.65 sone. The electric screwdriver had the lowest Loudness at 29.86 sone. The Sharpness (S) of the electric drill, the electric screwdriver, the angle grinder, the breaker, and the jackhammer were all above 2 acum. The breaker (2.77 vacil) and the concrete pump (3.21 vacil) had more prominent Fluctuation strength peak values (F_10_). The jackhammer (3.15 asper) and the breaker (3.81 asper) had higher Roughness(R).

**TABLE 6:** Results of physical and psychoacoustic indexes of ten typical noises.

**Mechanical sound**	**L_Aeq_/dB**	**L_5_/dB(A)**	**L_95_/dB(A)**	**L_5_–L_95_/dB(A)**	**N/sone**	**N_5_/sone**	**S/acum**	**S_5_/acum**	**F/vacil**	**F_10_/vacil**	**R/asper**	**R_10_/asper**
Angle grinder	89.35	90.99	87.67	3.32	63.68	67.17	2.76	3.07	0.91	2.09	1.40	1.93
Jackhammer	93.87	94.70	92.95	1.74	93.97	93.12	2.08	2.53	0.54	1.51	3.15	3.06
Electric drill	88.67	89.73	87.41	2.31	71.42	75.73	3.26	3.61	0.55	1.84	2.55	2.50
Electric screwdriver	75.82	76.37	75.28	1.08	29.86	30.69	2.90	3.07	0.57	1.85	1.49	1.99
Concrete vibrator	83.23	84.21	82.18	2.04	64.03	68.25	1.85	1.99	0.60	1.92	1.77	1.89
Loader	88.46	89.85	87.36	2.49	84.51	90.10	1.53	1.65	0.60	1.69	1.27	1.86
Dump truck	84.54	86.89	80.73	6.17	67.28	75.54	1.69	1.87	1.03	1.96	1.41	1.88
Excavator	79.34	80.34	77.95	2.39	52.25	54.51	1.68	1.86	0.66	2.00	1.37	1.92
Breaker	92.30	93.63	90.06	3.57	98.65	105.62	2.15	2.56	2.82	2.77	3.81	3.55
Concrete pump	86.31	87.67	84.94	2.73	76.08	80.42	1.54	1.66	0.74	3.21	1.24	1.77
Max	93.87	94.70	92.95	6.17	98.65	105.62	3.26	3.61	2.82	3.21	3.81	3.55
Min	75.82	76.37	75.28	1.08	29.86	30.69	1.53	1.65	0.54	1.51	1.24	1.77
Mean	86.19	87.44	84.65	2.78	70.17	74.12	2.14	2.39	0.90	2.08	1.95	2.24
SD	5.61	5.76	5.54	1.39	20.15	21.10	0.61	0.68	0.69	0.51	0.91	0.61

#### Annoyance Evaluation

The results for the evaluation of typical construction noise annoyance are shown in Table 7. ATCN_m_ represents the mean value of each noise annoyance rating of all respondents (95% confidence interval). Breaker, angle grinder and jackhammer had a mean annoyance greater than 4, which was a high level of annoyance. In particular, for the breaker, 82.7% of the responders thought it was “extremely annoying.” The lowest annoyance score was achieved by the electric screwdriver. Its average value was 1.83, which was close to “slightly annoying,” with 36.1% of the respondents thinking it was “not annoying at all.” The sound of dump truck operations, with an average of 2.52, scored between “slightly annoying” and “moderately annoying.” The mean annoyance of each of the other five noises was between 3 and 4, which was a medium annoyance level.

**TABLE 7:** Results of typical noise-induced annoyance.

**Annoyance/Values (N%)**	**1-Not annoying at all**	**2-Slightly annoying**	**3-Moderately annoying**	**4-very annoying**	**5-Extremely annoying**	**ATCNm (SD)**	**Zwicker’s PA**
Breaker	0	1 (0.8%)	3 (2.3%)	19 (14.3%)	110 (82.7%)	4.79 (0.508)	178.2
Angle grinder	0	2 (1.5%)	18 (13.5%)	46 (34.6%)	65 (48.9%)	4.36 (0.772)	82.79
Jackhammer	0	2 (1.5%)	20 (15%)	46 (34.6%)	65 (48.9%)	4.31 (0.780)	121.42
Electric drill	0	9 (6.8%)	47 (35.3%)	26 (19.5%)	51 (38.3%)	3.89 (1.002)	113.19
Concrete vibrator	0	10 (7.5%)	35 (26.3%)	62 (46.6%)	26 (19.5%)	3.78 (0.847)	77.7
Excavator	0	17 (12.8%)	41 (30.8%)	47 (35.3%)	28 (21.1%)	3.65 (0.955)	60.75
Concrete pump	0	25 (18.8%)	39 (29.3%)	35 (26.3%)	34 (25.6%)	3.59 (1.067)	86.6
Loader	0	30 (22.6%)	48 (36.1%)	36 (27.1%)	19 (14.3%)	3.33 (0.983)	95.97
Dump truck	4 (3.0%)	85 (63.9%)	26 (19.5%)	7 (5.3%)	11 (8.3%)	2.52 (0.958)	84.47
Electric screwdriver	48 (36.1%)	65 (48.9)	15 (11.3%)	5 (3.8%)	0	1.83 (0.774)	39.91

*N = 133.*

The relationship between ATCN_m_ and the physical and psychoacoustic indices of typical construction noise was analyzed. The results showed that the ATCN_m_ was significantly positively correlated with L_Aeq_, L_5_, L_95_, N, N_5_ (Table 8), from which it is clear that the noise intensity directly influenced the degree of annoyance. However, there was no correlation between the ATCN_m_ with S, F, and R. It can be seen that other psychoacoustic indices alone apart from Loudness could not represent noise annoyance. Based on Zwicker’s psychoacoustic annoyance (PA) model (Zwicker and Fastl, 1999), the PA of ten typical noise was calculated (See Table 7). The PA and ATCN_m_ showed a high degree of correlation according to a correlation analysis [*r*_(10)_ = 0.728 *p* = 0.017] (See Table 8). In addition, the correlation analysis between PA and 10 typical noises shows that except for S (when S < 1.75 acum, S does not participate in prediction in the model), the PA was significantly positively correlated with the model’s parameters N_5_, F, R.

**TABLE 8:** Correlation between ATCNm and Zwicker’s PA and typical noise acoustic indices.

		**L_Aeq_/dB**	**L_5_/ dB(A)**	**L_95_/ dB(A)**	**L_5_-L_95_/ dB(A)**	**N/sone**	**N_5_/ sone**	**S/acum**	**S_5_/ acum**	**F/vacil**	**F_10_/ vacil**	**R/asper**	**R_10_/ asper**	**Zwicker’s PA**
ATCN_m_	Correlation	0.775**	0.775*	0.790**	−0.023	0.718*	0.688*	0.000	0.117	0.436	0.233	0.605	0.588	0.728*
	Sig. (2-tailed)	0.008	0.012	0.007	0.950	0.019	0.028	0.999	0.747	0.208	0.517	0.064	0.074	0.017
Zwicker’s PA	Correlation	0.837**	0.830**	0.805**	0.221	0.883**	0.887**	0.001	0.130	0.744*	0.272	0.856**	0.859**	
	Sig. (2-tailed)	0.003	0.003	0.005	0.593	0.001	0.001	0.998	0.721	0.014	0.447	0.002	0.001	

*Correlation is significant at the 0.05 level (2-tailed).

**Correlation is significant at the 0.01 level (2-tailed).

Kendall’s *W* test (W^*a*^ = 0.558, *p* = 0.000) was used to evaluate the typical noise annoyance of 133 respondents, and principal components analysis was carried out on this basis. To identify the optimized components, varimax rotation was applied. Based on eigenvalues and scree plot analysis (eigenvalue = 0.972≈1, the scree plot showed an inflection point), three components (KMO = 0.735 > 0.7) were extracted, and they covered 57.9% of the total variance. Component 1 (C_1_) explained 22.1% of the variance in the data set, including the dump truck, the loader, the excavator and the concrete vibrator. Component 2 (C_2_) explained 20.2% of the variance in the data set, including the jackhammer, the angle grinder, the electric drill and the electric screwdriver. Component 3 (C_3_) explained 15.6% of the variance in the data set, including the breakers and the concrete pump (Table 9).

**TABLE 9:** Results of principal components analysis showing the classification results of ten typical noises.

	**Variance explained (%)**	**Mechanical sound**	**Components**		
			**1**	**2**	**3**
C1	22.1	Dump truck	*0.836*	0.086	−0.141
		Loader	*0.715*	0.199	0.317
		Excavator	*0.610*	0.077	0.548
		Concrete vibrator	*0.476*	0.231	0.150
C2	20.2	Jackhammer	0.106	*0.767*	0.214
		Angle grinder	0.022	*0.754*	−0.091
		Electric drill	0.144	*0.631*	0.144
		Electric screwdriver	0.346	*0.591*	−0.083
C3	15.6	Breaker	0.019	0.045	*0.869*
		Concrete pump	0.498	0.073	*0.529*

*Most important variables in each component are in Italic.*

**FIGURE 6:**
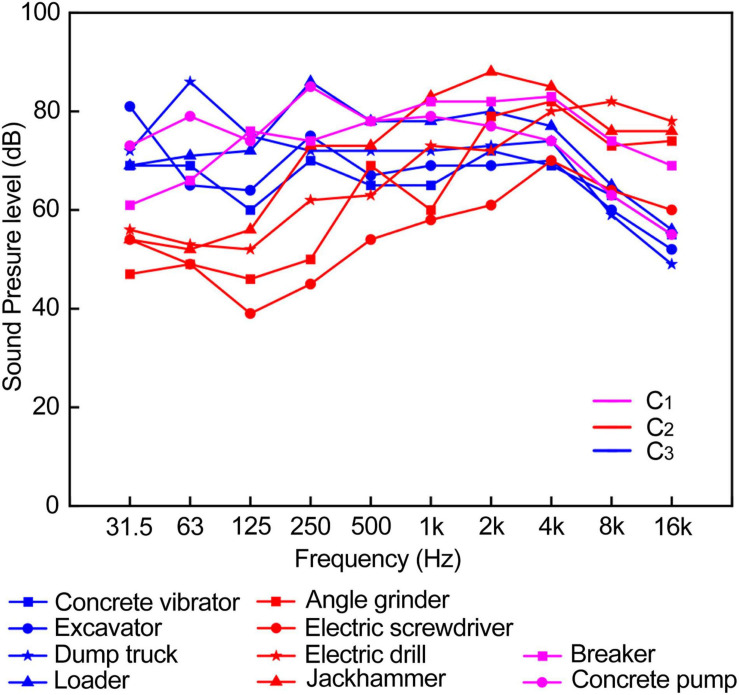
Frequency spectrums of 10 typical noise.

In order to explore the characteristics of mechanical noise contained in the three components, Frequency spectrums analysis was conducted on the ten typical noises through Constant Percentage Bandwidth (CPB) filters (one-third octave band) (Figure 6). Relative to C_2_, the mechanical noise in C_1_ and C_3_ was at a higher SPL in the low-frequency region below 500 Hz. The mechanical noise in C_2_ accounted for a relatively high proportion of medium- and high-frequency sound pressure levels of 1 kHz and above.

To further explore the characteristics of components, Jonckheere–Terpstra test was used to explore the difference between the three components in typical mechanical noise psychoacoustic indicators. The results showed that a significant difference was found in the Fluctuation strength among the three components, and F_C3_ > F_C2_ > F_C1_ (*z* = 2.137, *p* = 0.033), which indicated that the mechanical noise in C_3_ had the characteristics of high Fluctuation strength. Significant differences were also found in the Sharpness values of the three components, and S_C2_ > S_C3_ > S_C1_ (*z* = 2.324, *P* = 0.020), which indicated that the mechanical noise in C_2_ had high Sharpness.

Sharpness is a measure of spectral shape and refers to the proportion of high-frequency energy relative to the total energy (Zwicker and Fastl, 1999). It can be inferred from the frequency distribution of ten typical noise (See Figure 4), the high-frequency components of each mechanical noise in C_2_ accounted for a relatively high proportion, resulting in the sharp feeling of sound.

Temporal variations of sound resulted in two kinds of impressions: the Fluctuation strength and the Roughness (Rychtarikova and Vermeir, 2013). Fluctuation strength refers to the sound quality perceived when the individual Loudness fluctuations are audible (Hall et al., 2011). It gives people a sense of sound going up and down. The breakers and concrete pump in C_3_ group could reflect this feature (Figure 5): the sound fluctuation of the breaker about once every 1 s and the sound intensity change of the concrete pump at the 2nd second and the 7th second caused the feeling of sound fluctuation. This was the main reason that groups C_3_ and C_2_ were two different kinds of noise.

## Discussion

### “Cocktail Party Effect”

People can always grasp the information they want to know through hearing in a complex environment (Bronkhorst, 2015). This is the hearing selection ability of humans. It can allow them to recognize target sound information in a complex acoustic environment (Leech et al., 2009). Many factors will affect communications, such as the type of target sound, the spatial locality of the sound source, the level of interference sound, the level of background noise, and the person’s hearing ability (Bronkhorst, 2000). Based on this, in a noisy environment, speakers often increase the intensity of their voice to ensure the smooth progress of the communication (Castellanos et al., 1996). In addition, related studies have shown that cognitive factors such as working memory (WM) can help the brain better capture target information (Bidelman and Yoo, 2020). According to the results that affect the on-site communications (See Table 5), construction workers with a high degree of communications demand believe that post-specific noise has little effect on their communications and hearing. Perhaps there is a reasonable explanation that the high volume of communications and the familiarization to the on-site environment have improved the construction workers’ ability to capture the target sound, thereby making them believe that noise has a small impact on communications and hearing. Besides, for workers not equipped with radio communication devices, frequent communications needs force them to raise their voice when talking to each other. This consequently poses a potential threat to their voice. Other research has shown that for periodic noises like pulsed construction noises, there exist segmentations that lead to an audibly good separation of the speech signal and impulsive noise, and hence cause relatively little influence on the transmission of voice (Lee and Jeon, 2011). This is a likely explanation for why construction workers evaluate communications as a relatively small factor.

### Workers’ Unsafe Behavior

Most accidents are attributed to the unsafe behavior of the workers (Garavan and O’Brien, 2001). Excessive noise exposure is one of the factors that cause unsafe behavior (Kifle et al., 2014). According to the noise level measurement and typical noise collection, it is found that the noise exposure problems faced by construction workers cannot be ignored. Noise had a significant impact on the attention of construction workers, and a high-noise environment will accelerate their fatigue (Wang and Lv, 2015), thereby the probability of accidents is increased (Kirschenbaum et al., 2000).

According to the results of acoustic environment experience, almost all respondents believed that construction noise had an impact on communication. At the same time, they also believed that the on-site construction noise had an impact on their hearing. In other words, construction noise has a certain impact on the hearing and communications of construction workers. NIHL is mostly concentrated above 1000 Hz, which is also a critical range for workers to understand. This will result in reduced ability to perceive voice and warning signals (Suter, 2002). Due to the masking of noise, the workers cannot receive effective safety information (Deshaies et al., 2015), which leads to an increase in the probability of accidents. It is worth noting that the hearing protection device (HPD) will further reduce the workers’ auditory perception of the signal (Suter, 1992). This may reveal the reason why construction workers rarely wear HPD on their own initiative. This suggests that the hearing protection for construction workers should start with the control of the noise source.

### About Zwicker’s PA Model

Related studies believed that Zwicker psychoacoustic annoyance model could not be well applied to compare the annoyance degrees of tonal noises and atonal noises (Guo et al., 2016), and it ignored noise under transient variation (Park et al., 2015). In this study, the respondents’ self-evaluated degree of annoyance ATCNm for 10 typical construction noises had a significant correlation with Zwicker’s PA [*r*_(10)_ = 0.728 *p* = 0.017], which showed that Zwicker’s PA model was useful for evaluating the annoyance degree of construction noise and had a certain applicability. However, the correlation between the two and various psychoacoustic indicators was not consistent (See Table 8). This may be due to the small amount of typical noise samples collected and evaluated in this study, which did not reflect good statistical results. It may also be due to the high level of on-site construction noise intensity (L_Aeq_, as well as N) (See Table 6), which weakened the influence of psychoacoustic indicators. In future research, the noise sample size can be increased, and in-depth research can be carried out to determine the practicality of Zwicker’s PA model in evaluating the mechanical noise annoyance of construction sites, or to optimize the model in a targeted manner.

### Research Limitations

As part of the research, this article explored the subjective annoyance of construction workers to typical construction noise and its factor of acoustic characteristics. According to the results of on-site construction noise (See Figure 3), there were differences in the situation of on-site mechanical operations in different temporal stages, and the noise was not independently experienced by construction workers due to the combined operation of many machines in time and space. Research on the annoyance of construction workers caused by combined construction noise should be increased Related studies have shown that annoyance caused by combined noise is significantly higher than the annoyance caused by individual noise when L_Aeq_ in-creases over 65 dB(A) (Lee et al., 2015). In this study, respondents’ subjective evaluation of typical noise annoyance was based on their long-term experience of the noise in their work experience, while the audio representative of typical construction noise was the standard audio collected on site. Compared with the laboratory listening evaluation, the correspondence between the evaluation results and the evaluation objects was weakened, but the results were more practical. In addition, unfortunately, due to the environmental conditions and safety management, the standardization of sound acquisition is limited, which is the limitation of this research.

## Conclusion

This research conducted a 30-week construction site noise monitoring to summarize the noise level, collected 10 typical construction noises, analyzed their physical and psychoacoustic characteristics, and obtained objective data on the occupational environmental noise of construction workers. A questionnaire survey of 133 construction workers of different types of work was conducted to determine the impact of construction noise. The results showed that the noise situation on construction sites is not optimistic, and the construction workers have been affected to varying degrees in terms of psychological experience, hearing ability, and on-site communications. The respondent’s post-specific noise level was significantly positively correlated with the evaluation of the noisiness. The self-evaluated impact on hearing ability was significantly positively correlated with the post-specific noise level and was significantly negatively correlated with the demand for communications. The impact on on-site communications was significantly positively correlated with the respondent’s post-specific noise level and their age, and significantly negatively correlated with the demand for communications. In addition, correlation analysis and cluster analysis both showed that the annoyance caused by typical construction noise was correlated to its physical and psychoacoustic characteristics. These subjective and objective data and their correlation provided a basis for strengthening on-site management, implementing site noise reduction, optimizing construction machinery, and providing hearing protection for construction workers.

## Data Availability Statement

The original contributions presented in the study are included in the article/supplementary material, further inquiries can be directed to the corresponding author/s.

## Ethics Statement

Ethical review and approval was not required for this study in accordance with the local legislation and institutional requirements. The participants provided written informed consent to participate in the study.

## Author Contributions

XY: noise collection and acoustic analysis, questionnaire design, and manuscript writing. YW: site noise measurement and questionnaire survey. RZ: statistical analysis of the data. YZ: overall supervision, research design, and the structuring of the manuscript. All authors contributed to the article and approved the submitted version.

## Conflict of Interest

YW is employed by the Railway No.9 Bureau Group 4th Engineering Co., Ltd., China. The remaining authors declare that the research was conducted in the absence of any commercial or financial relationships that could be construed as a potential conflict of interest.

## Publisher’s Note

All claims expressed in this article are solely those of the authors and do not necessarily represent those of their affiliated organizations, or those of the publisher, the editors and the reviewers. Any product that may be evaluated in this article, or claim that may be made by its manufacturer, is not guaranteed or endorsed by the publisher.
